# An Atypical Manifestation of Cellulitis Induced by Proteus mirabilis

**DOI:** 10.7759/cureus.85249

**Published:** 2025-06-02

**Authors:** Mojir Muhajir, Jamie McDermott, Veronica Rodriguez

**Affiliations:** 1 Medical School, Midwestern University Arizona College of Osteopathic Medicine, Glendale, USA; 2 Family Medicine, HealthyU Clinics, Phoenix, USA

**Keywords:** amoxicillin-clavulanate, augmentin, cellulitis, gram negative, p. mirabilis, proteus mirabilis, soft tissue infection, susceptibility

## Abstract

Cellulitis is a common bacterial skin infection typically caused by organisms such as *Streptococcus* and *Staphylococcus* species. *Proteus mirabilis *(*P. mirabilis*), a gram-negative rod more often linked to urinary tract infections, is not a frequent cause of cellulitis. However, certain biological traits like motility and the ability to form biofilms can occasionally contribute to infections in compromised tissue. This case discusses an uncommon presentation of cellulitis involving *P. mirabilis* and its clinical management. An 81-year-old woman with a history of vascular disease and other chronic conditions presented with a red, swollen wound on her lower leg after a minor injury. Initial treatment with empiric antibiotics did not resolve the symptoms, and wound cultures later revealed *P. mirabilis.* Treatment was adjusted to amoxicillin-clavulanate, but persistent inflammation required a second course of antibiotics due to the patient’s sulfa allergy. The infection eventually resolved without systemic complications. Although not a typical skin pathogen, *P. mirabilis* may occasionally be found in such infections, particularly when initial therapies are ineffective. This case emphasizes the importance of considering less common organisms in cellulitis cases that do not improve with standard treatment. Tailoring antibiotics based on culture results is essential to achieving resolution and avoiding complications. A broad diagnostic approach can be valuable in managing unusual or resistant infections.

## Introduction

Cellulitis is a prevalent bacterial skin infection characterized by progressive inflammation, erythema, tenderness, and potential lymphatic spread. In adults, the most frequently implicated pathogens are* Streptococcus* species and* Staphylococcus aureus* [1]. Other pathogens, such as *Proteus mirabilis* (*P. mirabilis*), are more commonly associated with urinary tract infections (UTIs). *P. mirabilis* is not typically considered a cutaneous pathogen; it originates from the gastrointestinal tract and is known for its strong urothelial adherence [2,3]. Although it can cause skin infections in immunocompromised individuals or through wound contamination, its virulence factors are less adapted for cutaneous spread [4]. Its pathogenicity is attributed to swarming motility, biofilm formation, and urease production [5]. This report aims to broaden the understanding of *P. mirabilis* as a potential etiological agent in cellulitis and to discuss its clinical implications.

## Case presentation

An 81-year-old female patient with a medical history of peripheral vascular disease, hypertension, hyperlipidemia, and rheumatoid arthritis presented to the family medicine clinic with swelling and erythema of the left lower leg. Two weeks prior, she had sustained minor trauma to the area after striking it against a stainless steel dishwasher. She attempted supportive care at home with limited improvement, including alternating heat and ice, limb elevation, and bandaging with an all-cotton elastic (ACE) wrap. She denied systemic symptoms such as fever, chills, headache, muscle weakness, or wound drainage.

On physical examination, there was localized erythema, pitting edema, and tenderness over the anterior aspect of the left lower leg, without purulence or signs of systemic infection. These findings suggested a less severe or typical skin infection, rather than one involving methicillin-resistant *Staphylococcus aureus* (MRSA). Laboratory testing, including complete blood count, was within normal limits, and no leukocytosis was observed. Repeat labs were not obtained, and the patient was followed clinically. Imaging was deferred, and empiric treatment with cephalexin 500 mg orally four times daily was initiated. Sensitivity analyses were not performed. The patient was counseled on wound care and advised to monitor for worsening symptoms.

**Figure 1:**
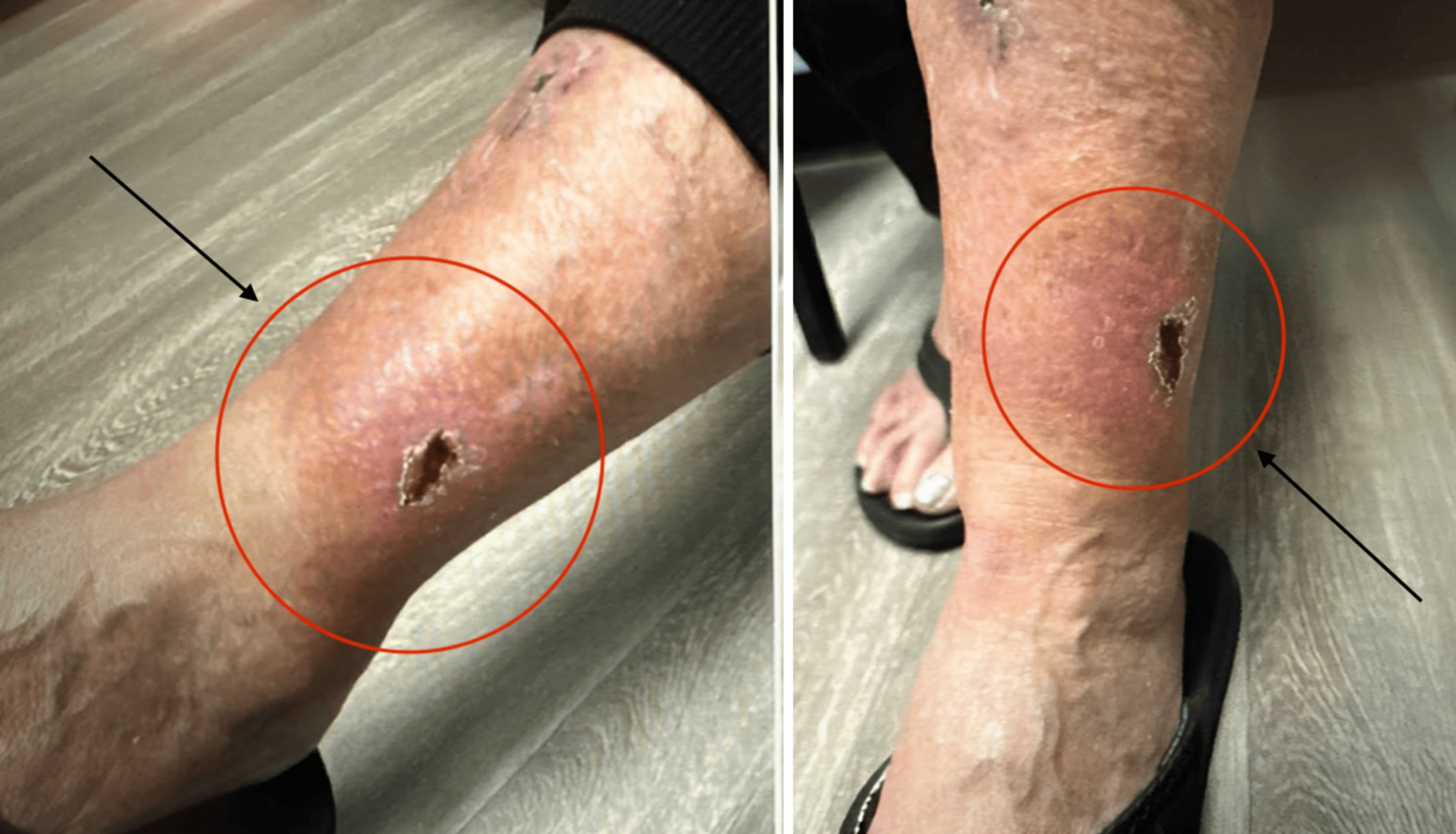
Left leg cellulitis Patient presented with unchanged lower extremity edema with signs of cellulitis and possible ulceration after initial 10-day treatment with amoxicillin-clavulanate.

Superficial wound cultures collected before antibiotic therapy subsequently grew* Proteus mirabilis*. Cephalexin was discontinued, and the patient was prescribed amoxicillin-clavulanate 875/125 mg orally twice daily for 10 days. At follow-up, she reported ongoing swelling and erythema without new symptoms (Figure 1). Although trimethoprim-sulfamethoxazole is commonly effective against *P. mirabilis*, its use was avoided due to the patient’s reported sulfonamide allergy. A second 10-day course of amoxicillin-clavulanate was prescribed. Clinical improvement was noted with continued close outpatient follow-up.

## Discussion

**Figure 2:**
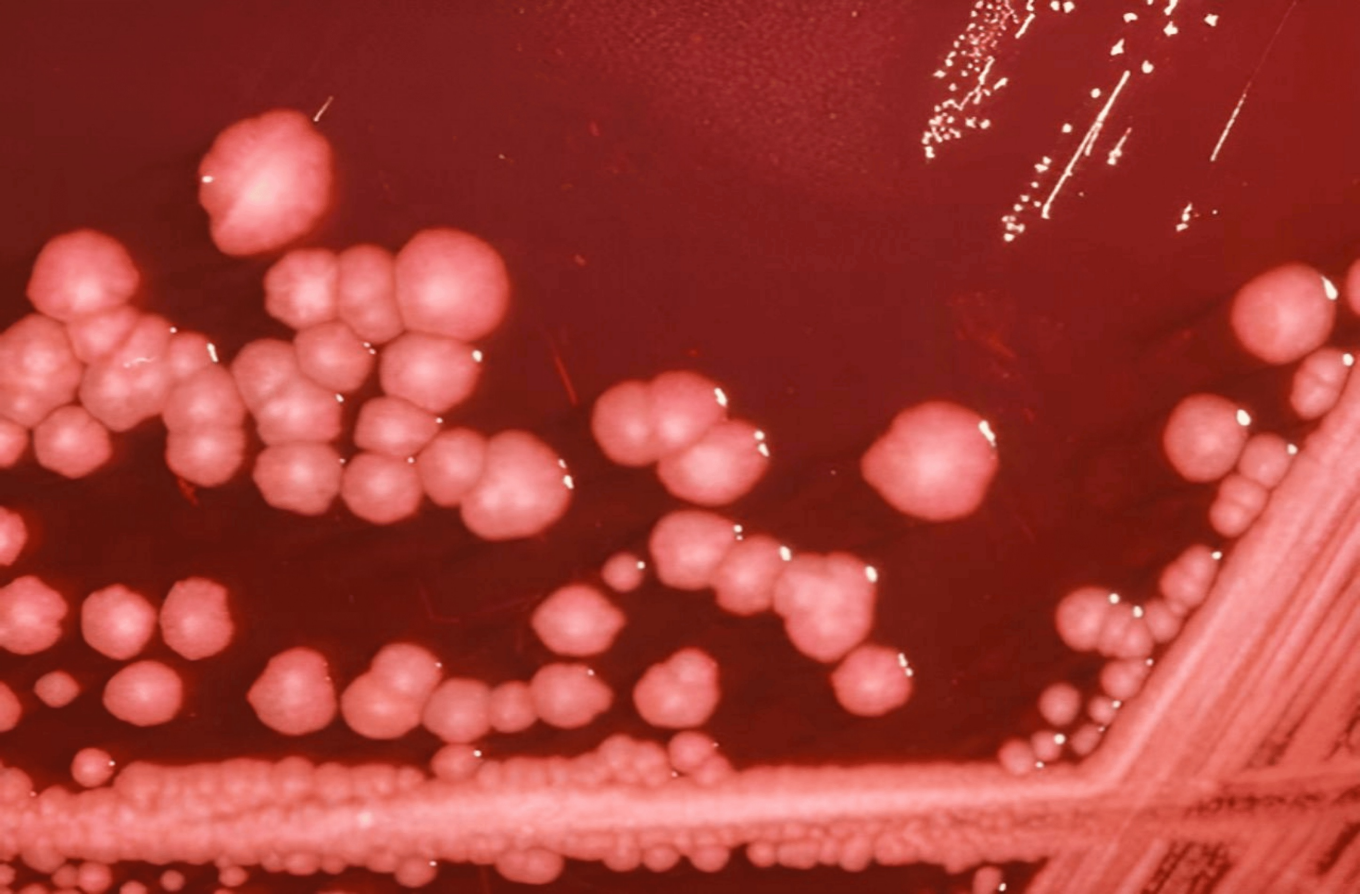
Biofilm formation by Proteus mirabilis on blood agar. The image displays the characteristic clustered, mucoid colonies consistent with biofilm production. P. mirabilis forms robust biofilms on biotic and abiotic surfaces, contributing to its persistence in chronic infections, resistance to antibiotics, and evasion of host immune responses. Image credit: "Proteus mirabilis colonies on XLD agar" by the Centers for Disease Control and Prevention (CDC), Public Health Image Library (PHIL #6691). As a work of the U.S. federal government, this image is in the public domain [10].

*Proteus mirabilis* is a motile, facultatively anaerobic gram-negative rod commonly associated with urinary tract infections, particularly in individuals with indwelling catheters or structural abnormalities of the urinary tract [6]. It belongs to the family* Enterobacteriaceae *and is distinguished by its production of urease, which hydrolyzes urea into ammonia and carbon dioxide, raising the pH of urine and predisposing to the formation of struvite stones [7]. The organism is equipped with peritrichous flagella that enable its characteristic “swarming motility” on agar plates and across mucosal surfaces, facilitating colonization and tissue invasion [8,9]. In addition to swarming, *P. mirabilis* exhibits robust biofilm-forming capacity on both biological tissues and medical devices, which contributes to its persistence and resistance to host defenses and antibiotics (Figure 2) [10,11]. 

Though primarily associated with urinary tract infections, *P. mirabilis* can act as an opportunistic pathogen in a variety of compromised tissues, including skin wounds and areas of poor perfusion. While not typically considered a common cause of skin and soft tissue infections (SSTIs), *P. mirabilis* has been identified in a small but notable subset of such cases. One retrospective study reported its presence in only 1.2% of SSTIs, highlighting its rarity as a monomicrobial cause [2]. However, other investigations have observed its involvement more frequently in polymicrobial infections, particularly those involving chronic wounds in patients with underlying comorbidities such as diabetes mellitus or peripheral vascular disease [3]. These conditions often impair local immune defenses and wound healing capacity, creating an environment where less common pathogens like *P. mirabilis* can thrive [11]. This pattern suggests that *P. mirabilis* may serve as an opportunistic organism by exploiting ischemic or necrotic tissue, especially in elderly patients or those with chronic health issues, to establish infection.

Balancing the treatment of common versus atypical pathogens presents an ongoing challenge in clinical practice. While empiric therapy is typically guided by the most likely causative organisms, the persistence or progression of infection despite appropriate initial treatment should prompt clinicians to revisit the differential diagnosis and obtain microbiologic cultures to identify less common pathogens. Infections caused by *P. mirabilis* require antibiotic selection guided by culture and sensitivity data, as resistance patterns can vary. First-line treatment options generally include trimethoprim-sulfamethoxazole, ciprofloxacin, or ampicillin, depending on susceptibility results [9-14]. However, therapeutic decisions must also take into account individual patient factors. In this case, the patient's reported sulfonamide allergy eliminated trimethoprim-sulfamethoxazole as a safe option because of the potential risk of severe hypersensitivity reactions such as Stevens-Johnson Syndrome [15]. Consequently, the clinical team opted to continue treatment with amoxicillin-clavulanate, which remained a reasonable alternative given the organism’s susceptibility profile. Ongoing monitoring of the patient’s clinical response and laboratory markers was used to assess the effectiveness of this regimen. Additional adjustments to therapy were considered based on the patient's progress and any changes in clinical status.

## Conclusions

This case highlights the critical need to maintain a broad and evolving differential diagnosis in patients presenting with cellulitis that does not respond to initial empiric antibiotic therapy. While common pathogens such as S*treptococcus* and S*taphylococcus *are often at the forefront of clinical suspicion, this case demonstrates that less typical organisms, like *Proteus mirabilis*, can occasionally be the underlying cause, especially in patients with compromised vascular supply, chronic wounds, or advancing age. These populations may present with atypical infection patterns due to altered immune responses and tissue environments. Recognizing such possibilities early in the clinical course is vital, as delayed identification of the causative organism can lead to prolonged morbidity, ineffective treatment, and increased healthcare utilization. Timely acquisition of wound cultures and implementation of susceptibility-guided antibiotic therapy are essential not only for tailoring treatment but also for limiting unnecessary broad-spectrum antibiotic use and curbing resistance. Ultimately, this case reinforces the value of remaining vigilant to unusual etiologies in refractory infections, advocating for a dynamic, individualized approach to diagnosis and management that prioritizes both clinical judgment and microbiological evidence.
